# Adiponectin as a novel biomarker of disease severity in alopecia areata

**DOI:** 10.1038/s41598-021-92853-1

**Published:** 2021-07-05

**Authors:** Anna Stochmal, Anna Waśkiel-Burnat, Sylwia Chrostowska, Michał Zaremba, Adriana Rakowska, Joanna Czuwara, Lidia Rudnicka

**Affiliations:** Department of Dermatology, Medical University of Warsaw, Warsaw, Poland

**Keywords:** Immunology, Biomarkers, Diseases, Medical research

## Abstract

The frequent coexistence of obesity and metabolic syndrome in patients with alopecia areata may indicate the common pathogenetic pathway in these conditions with an important role of adipokines. The aim of the study was to evaluate the serum level of adiponectin, resistin and leptin in patients with alopecia areata in comparison to healthy controls. The study included 65 patients with alopecia areata and 71 healthy controls. The concentration of adipokines was determined with the enzyme-linked immunosorbent assay. The mean concentrations of adiponectin and resistin were significantly lower in the sera of patients with alopecia areata when compared to healthy controls (7966 $\pm$ 4087 vs 9947 $\pm$ 5692 ng/ml; p = 0.0312 and 11.04 $\pm$ 3.88 vs 14.11 $\pm$ 8.69 ng/ml; p = 0.0176, respectively). A negative correlation between the serum level of adiponectin and severity of alopecia tool (SALT) score was observed (r = − 0.26; p < 0.05). The concentration of adiponectin was significantly lower in patients with alopecia universalis than in patients with patchy alopecia areata (4951 $\pm$ 2499 vs 8525 $\pm$ 4085 ng/ml; p = 0.0135). No significant difference in the serum concentration of leptin was observed between patients with alopecia areata and healthy controls. The negative correlation between the serum level of adiponectin and hair loss severity indicates that adiponectin may be considered a marker of hair loss severity in alopecia areata. Further studies are needed to evaluate the role of resistin in patients with alopecia areata and its decreased level irregardless of severity or activity of the disease.

## Introduction

Adipose tissue is no longer considered as an inert tissue used mianly for energy storage. Adipocytes actively produce and secrete proteins, adipokines, with metabolism regulating properties. They are involved in the regulation of energy expenditure, insulin sensitivity, glucose and lipid metabolism, as well as endothelial function, playing an important role in the pathogenesis of metabolic syndrome. What is more interesting, adipokines also emerge as active regulators of physiologic and pathologic processes, including immunity and inflammation^1^. They are characterized by pro- or anti-inflammatory properties. The balance of pro- and anti-inflammatory adipokines is maintained in individuals with normal metabolic status. In cases of calorie restriction and starvation the concentration of pro-inflammatory adipokines is decreased and the level of anti-inflammatory adipokines is elevated, which is a signal of energy deficit that contributes to immune suppression. In obesity, pro-inflammatory adipokines are predominate^2^. Recently, the role of adipokines has been described in the development of autoimmune and inflammatory disorders including rheumatoid arthritis, systemic lupus erythematosus, psoriasis and inflammatory bowel diseases^3,4^.

The melanin biosynthesis pathway was described in the adipose tissue^5^. Specifically, both melanin and the components of melanin biosynthesis were observed in the adipose tissue. They were expressed at much higher levels in samples from the obese individuals. Both melanin and components of the melanogenic pathway have anti-inflammatory and antioxidant properties. Thus, it may be hypothesized that they help to delay the onset of obesity-related conditions linked to the increased inflammation and oxidative stress^6^. In the absence of melanin, dietary factors, hypoxia and the peroxidation of cellular lipids generate the excess of reactive oxygen species in adipocytes, leading to apoptosis. It results in the release of pro-inflammatory and the deficiency of anti-inflammatory adipokines^6^.

Follicular melanocytes, similarly to epidermal melanocytes, originate from neural crest, but they are characterized by a larger size and longer dendrites^7,8^. They reside at the basal layer of the hair matrix, in close proximity to the dermal papilla, and tend to rest on the basal lamina. The expression of proopiomelanocortin (POMC) gene, production of the POMC peptides (β-endorphin, ACTH, α-MSH) and finally melanogenesis are synchronized with hair follicle cycle^7,9^. They are lowest in telogen (resting phase), increases during anagen (growing phase), and decreases in catagen (involution phase)^8,10^.

Alopecia areata is an autoimmune form of non-scarring hair loss that may affect any hair-bearing area^11^. The prevalence of the disease varies between 0.1 and 0.2% in the general population^12,13^. The pathogenesis of alopecia areata has not been fully elucidated. However, numerous studies indicate that alopecia areata is associated with systemic autoimmune activation implying significantly elevated serum levels of Th1 (IL-1β, IL-2, IL-12, TNF-α, and IFN-γ), Th2 (IL-4, IL-10, IL-13, IL-25, IL-31) and Th17 cytokines (e.g. IL-17A)^14^. It has been hypothesized that melanocyte-derived autoantigen is the target of immune response in alopecia areata^15^. Importantly, in patients with alopecia areata hair loss is limited to pigmented hairs in anagen phase^16^. A tendency for the initial regrowth of white hairs is observed^17^. An association between alopecia areata and vitiligo is reported^18^. Finally, melanocytes are a significant component of the hair bulb, which is the site of immune attack in alopecia areata^17^. To date, limited reports concerning the role of adipokines in alopecia areata have been published.

## Objective

The aim of the study was: (1) to evaluate the serum levels of adipokines, such as adiponectin, resistin and leptin in patients with alopecia areata and compare to healthy controls; (2) to analyze correlations between the levels of adipokines and the severity of hair loss and compare the levels of adipokines between patients with patchy alopecia areata, areata totalis and alopecia universalis; (3) to compare the levels of adipokines between patients with active, stable and remitting alopecia areata; (4) to analyze correlations between the levels of adipokines and the duration of hair loss and (5) to assess correlations between the levels of adipokines, age and body mass index (BMI) of the patients with alopecia areata.

## Material and methods

### Patients

The study included 65 patients with alopecia areata and 71 healthy controls matched for age, sex and BMI. The diagnosis of alopecia areata was established based on a detailed medical history, clinical examination and trichoscopy. In patients with alopecia areata, the severity of hair loss was assessed with the severity of alopecia tool (SALT)^19^. Based on the severity of hair loss, the patients were divided into three groups: (1) patients with patchy alopecia areata (partial hair loss), (2) patients with alopecia totalis (complete scalp hair loss) and (3) patients with alopecia universalis (complete scalp and body hair loss). The activity of hair loss was evaluated as follows: (1) progressive alopecia areata, an increase in total hair loss of more than 5%; (2) stable, a change in total hair loss of less than 5%; (3) remitting alopecia areata, a decrease in total hair loss of more than 5% over the month prior to the laboratory tests^20^.

Clinically ambiguous cases as well as patients with a history of using any treatment that could impact the metabolic status within 3 months prior to the examination, patients with other autoimmune diseases, pregnant women and patients under the age of 18 were excluded from the analysis. All patients were examined in the outpatient clinic between September and October 2020.

The study protocol conformed to the principles of the World Medical Association’s Declaration of Helsinki and was approved by the Medical University of Warsaw Review Board for Ethics in Human Research (protocol number KB/142/2020). Written informed consent was obtained from all participants of the study.

### Biochemical measurements

All blood samples (6 ml each) were collected to obtain the serum for further analysis. Frozen serum samples were stored at − 80 °C. The serum concentrations of adiponectin, leptin and resistin were measured to assess metabolic disturbances. The serum concentrations of adiponectin and resistin were evaluated in 65 patients with alopecia areata and 71 healthy controls, while the serum concentration of leptin was measured in 55 patients with alopecia areata and 45 healthy controls. The concentrations of all proteins were assessed with the enzyme-linked immunosorbent assays (Quantikine ELISA Kits R&D Systems, Minneapolis, USA) according to the protocol provided by the manufacturer.

### Statistical analysis

The Shapiro–Wilk test was used to assess the normality of data distribution. To compare differences between the groups, the Student’s t test was used for data with normal distribution and the Mann–Whitney U test for non-parametric variables. The Spearman’s rank test was used to measure correlations between variables (r). Data were considered significant for p < 0.05. The analyses were performed with Statistica 13.3 (StatSoft/TIBCO, Cracow, Poland).

## Results

### Patients

The study included 65 patients with alopecia areata. The severity of hair loss evaluated with SALT varied between 2 and 100% (mean SALT score: 30%). Patchy alopecia areata was observed in 51 (78%) patients, alopecia totalis in 7 (11%) patients and alopecia universalis in 7 (11%) patients. Active hair loss was observed in 21 (32%) patients. Stable disease was diagnosed in 25 (39%) cases, while remitting alopecia areata in 19 (29%) patients. The control group consisted of 71 healthy individuals matched for age, sex and BMI. Two patients with alopecia areata and one individual from the control group were diagnosed with metabolic syndrome. One patient with alopecia areata was diagnosed with diabetes mellitus type 1, whereas none of healthy controls had a history of diabetes. There was no significant differences in mean concentrations of fasting glucose or lipids between patients with alopecia areata and healthy controls (p > 0.05).

Detailed characteristic of patients with alopecia areata and healthy controls is presented in Table 1.

**Table 1:** Characteristic of patients with alopecia areata and healthy controls.

Parameter	Patients with AA (n = 65)	Healthy controls (n = 71)
Age, mean ± SD (years)	44 ± 17	46 ± 19
Sex (women/men)	49/16	53/18
BMI, mean ± SD (kg/m^2^)	24.78 ± 2.19	25.67 ± 3.21
Cholesterol, mean ± SD (mg/dl)	183.5 ± 20.5	179 ± 19
Low-density lipoprotein, mean ± SD (mg/dl)	109.5 ± 7.5	103 ± 6.5
High-density lipoprotein, mean ± SD (mg/dl)	65 ± 1	55 ± 1
Triglycerides, mean ± SD (mg/dl)	114.5 ± 21.5	123 ± 25.5
Fasting glucose, mean ± SD (mg/dl)	93 ± 17.5	96 ± 19.5
Duration of the episode of alopecia, mean ± SD (years)	3.55 ± 1.63	NA
SALT score, mean ± SD (%)	30 ± 37	NA
**Severity of the disease (n)**		NA
Patchy	51	
Totalis	7	
Universalis	719	
**Activity of the disease (n)**		NA
Active^a^	21	
Stable^b^	25	
Remitting^c^	19	

*BMI* body mass index, *SALT* severity of alopecia tool, *SD* standard deviation, *AA* alopecia areata, *NA* not applicable.

^a^Active alopecia areata—an increase in total hair loss of more than 5% during 1 month prior to laboratory tests.

^b^Stable alopecia areata—a change in total hair loss of less than 5% during 1 month prior to the laboratory tests.

^c^Remitting alopecia areata—a decrease in total hair loss of more than 5% during 1 month prior to the laboratory tests.

### Biochemical measurements

**Figure 1:**
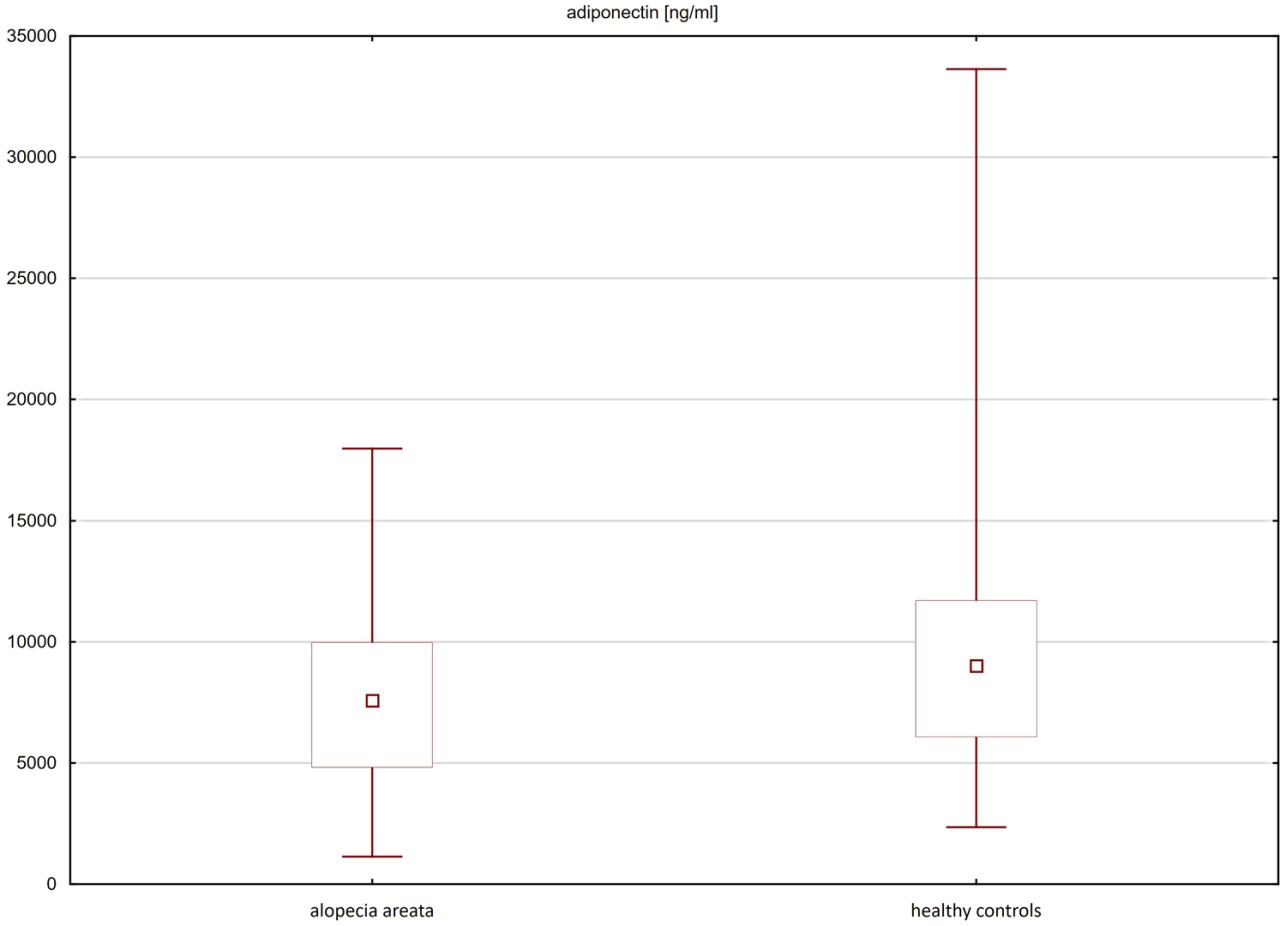
Serum concentrations of adiponectin in patients with alopecia areata and healthy controls.

**Figure 2:**
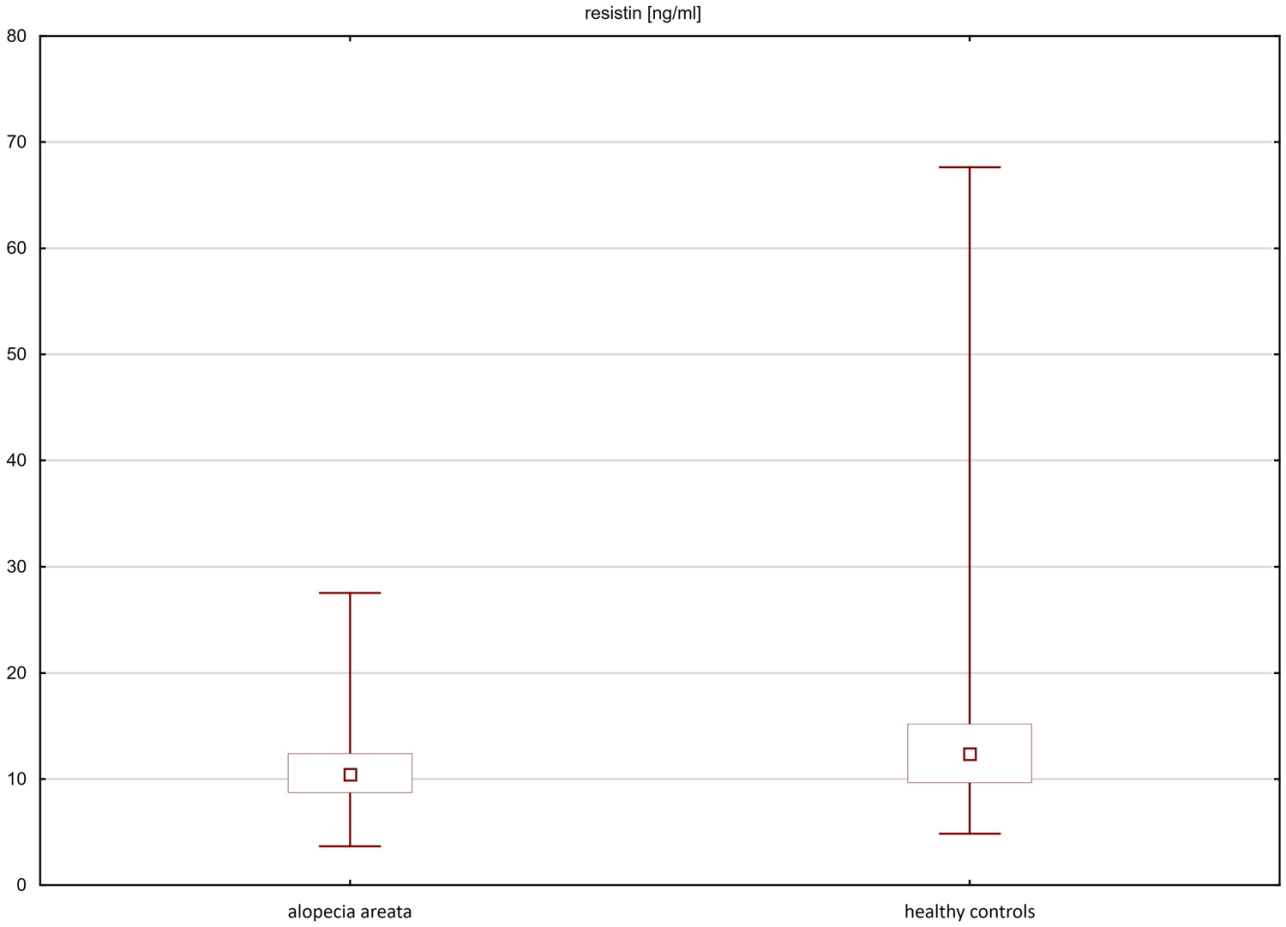
Serum concentrations of resistin in patients with alopecia areata and healthy controls.

The mean concentrations of adiponectin (7966 $\pm$ 4087 ng/ml vs 9947 $\pm$ 5692 ng/ml; p = 0.0312) and resistin (11.04 $\pm$ 3.88 ng/ml vs 14.11 $\pm$ 8.69 ng/ml; p = 0.0176) were significantly lower in the sera of patients with alopecia areata compared to healthy controls. No significant differences were observed in the serum concentrations of leptin in patients with alopecia areata and healthy controls (Table 2; Figs. 1, 2).

**Table 2:** Serum concentrations of adiponectin, resistin and leptin in patients with alopecia areata and healthy controls.

Parameter (mean ± SD)	Patients with AA (n = 65)	Healthy controls (n = 71)	p value
Adiponectin (ng/ml)	7966 ± 4087	9947 ± 5692	0.0312
Resistin (ng/ml)	11.04 ± 3.88	14.11 ± 8.69	0.0176
Leptin (pg/ml)	17683 ± 18048	12789 ± 11492	0.2658

*SD* standard deviation, *AA* alopecia areata.

**Table 3:** Differences in the serum concentrations of adiponectin and resistin between patients with patchy alopecia areata, alopecia totalis and alopecia universalis.

Parameter (mean ± SD	Patients with PAA (n = 51)	Patients with AT (n = 7)	p value	Patients with AA (n = 51)	Patients with AU (n = 7)	p value	Patients with AT (n = 7)	Patients with AU (n = 7)	p value
Adiponectin (ng/ml)	8525 ± 4085	6910 ± 4278	0.3119	8525 ± 4085	4951 ± 2499	0.0135	6910 ± 4278	4951 ± 2499	0.5349
Resistin (ng/ml)	11.08 ± 4.46	11.98 ± 4.30	0.5428	11.08 ± 4.46	9.77 ± 4.08	0.3597	11.98 ± 4.30	9.77 ± 4.08	0.2086

*SD* standard deviation, *PAA* patchy alopecia areata, *AT* alopecia totalis, *AU* alopecia universalis.

### Correlations between serum concentrations of adipokines and selected clinical parameters

**Figure 3:**
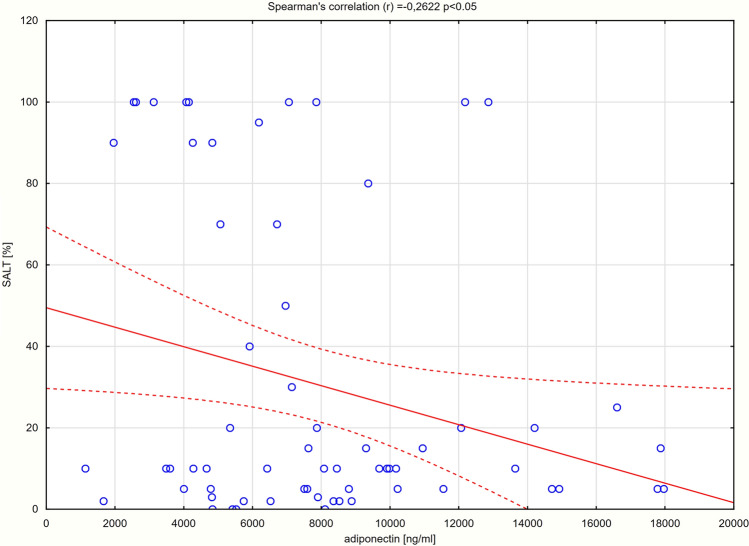
A correlation between the serum concentration of adiponectin and the severity of alopecia areata.

A. Disease severity
  A statistically significant negative correlation was detected between the serum concentrations of adiponectin and SALT score (r = − 0.26; p < 0.05) (Fig. 3).
  Moreover, the concentration of adiponectin was significantly decreased in patients with alopecia universalis compared to patients with patchy alopecia areata (4951 $\pm$ 2499 vs 8525 $\pm$ 4085 ng/ml; p = 0.0135). Statistically significant differences between the serum levels of adiponectin in patients with patchy alopecia areata and alopecia totalis or between patients with alopecia universalis and totalis were not demonstrated (p > 0.05) (Table 3). No significant correlation was observed between the serum concentrations of resistin and SALT score (r = 0.07; p > 0.05) (Suppl. Figure 1). Moreover, no significant differences in the serum levels of resistin between patients with patchy alopecia areata, alopecia totalis and universalis were observed (p > 0.05) (Table 3).
B. Disease activity
  There were no significant differences between the serum levels of adiponectin and resistin in patients with active hair loss compared to patients with stable (p = 0.1275 and p = 0.5235, respectively) and remitting alopecia areata (p = 0.9886 and p = 0.5695, respectively) (Suppl. Table 1).
C. Duration of hair loss, age, BMI

No significant correlations were observed between the serum concentrations of adiponectin and resistin and the duration of hair loss (p > 0.05) (Suppl. Figures 2, 3). Moreover, there were no correlations between the serum levels of adipokines, age and BMI of the patients with alopecia areata (p > 0.05).

## Discussion

Alopecia areata is sometimes considered solely as a cosmetic problem. However, it was described that alopecia areata might be associated with an increased risk of the development of inflammatory and metabolic comorbidities^21^. In a study performed by Conic et al.^22^ hyperlipidemia (19.8% vs 6.6%), obesity (18.1% vs 3.0%), diabetes mellitus (11.4% vs 7.4%) and metabolic syndrome (1.4% vs 0.3%) were more frequently reported in patients with alopecia areata compared to healthy controls. Indeed, the serum level of insulin, C-peptide and homeostasis model assessment for insulin resistance (HOMA-IR) were significantly higher in patients with alopecia areata compared to controls^23,24^. Conversely, the lipid profiles including high-density lipoprotein cholesterol (HDL), low-density lipoprotein (LDL) cholesterol, very low-density lipoprotein (VLDL) cholesterol and triglycerides were similar to those in healthy controls^23^.

The common presence of metabolic abnormalities in patients with alopecia areata and the fact that melanocytes are a possible target of activated cells in alopecia areata suggest that adipokines may play a role in the pathogenesis of the disease. However, to date limited number of studies has evaluated the role of adipokines in alopecia areata.

Adiponectin is a serum protein produced mainly by the adipose tissue. However, other cells such as the endothelial cells, fibroblasts, leukocytes and macrophages may also synthesize adiponectin^25^. Adiponectin has an anti-inflammatory effect and reduces T cell responsiveness, B cell lymphopoiesis, and TNF-α synthesis. It also promotes IL-10 production. The decreased serum levels of adiponectin compared to healthy controls were reported in patients with psoriatic arthritis, Sjogren’s syndrome and multiple sclerosis^26^. In the present study lower serum levels of adiponectin were revealed compared to those in healthy individuals. Moreover, a negative correlation was identified between the serum levels of adiponectin and disease severity and the lowest concentration of this adipokine was indicated in patients with alopecia universalis. However, the present study showed that the concentration of adiponectin did not correspond to the activity of the disease.

Resistin is a pro-inflammatory protein involved in human lipid, glucose and insulin homeostasis. It is primarily secreted by macrophages, not adipocytes, so it may have a direct impact on inflammatory processes^25^. Resistin binds toll-like receptor 4 (TLR4) on human leukocytes leading to the production of pro-inflammatory cytokines such as IL-6, IL-23, and IL-1β. In vitro treatment with resistin triggers pro-inflammatory response from adipocytes by the secretion of TNF-α, IL-6, MCP-1 and peripheral blood mononuclear cells by production of TNF-α, IL-6 and IL-1β^26^. The increased serum concentrations of resistin and a positive correlation between the levels of resistin and the activity of inflammatory processes were observed in numerous autoimmune and inflammatory disorders, such as rheumatoid arthritis, multiple sclerosis, psoriasis, systemic lupus erythematosus, systemic sclerosis, Sjogren’s syndrome and inflammatory bowel diseases^3,25,26^. To date, the role of resistin has not been confirmed in alopecia areata. In present study the level of resistin observed in patients with alopecia areata was decreased compared to healthy controls. No association was present between serum resistin levels and hair loss activity and severity. In patients with alopecia areata the lower serum level of resistin, which is considered as a molecule aggravating inflammation, may be explained by its local pro-inflammatory action being independent from the systemic concentration.

Leptin is a peptide hormone which acts via the OB-Rb leptin receptor coupled to the JAK/STAT signaling pathway^27^. It is considered as a critical regulator of body weight by promoting satiety and increasing energy consumption^26^. Leptin is mainly produced by the white adipose tissue. Positive correlations were described between the serum concentrations of leptin and the amount of adipose tissue and body mass index^27^. Moreover, leptin is a pro-inflammatory mediator. It induces the production of pro-inflammatory cytokines, such as TNF-α and IL-6, and reactive oxygen species in cultured monocytes. Leptin also stimulates the production of chemokines by macrophages and alters Th1/Th2 balance in favor of Th1 phenotype^27^. Despite the probable involvement of leptin in the pathogenesis of several autoimmune diseases, no differences between the serum concentrations of leptin in patients with alopecia areata and healthy controls were detected in present study.

On the contrary to presented findings, previous report on serum adipokines level performed by Serarslan et al.^28^ did not show significant differences in adiponectin and leptin concentration in patients with alopecia areata compared to healthy controls. Higher serum levels of both adipokines were found in patients with scalp hair loss in comparison to patients with isolated beard and eyebrow alopecia areata.

To summarize, abnormalities in the synthesis of adipokines may indicate that adipose tissue participates in the development and progression of alopecia areata. In particular, impaired adiponectin levels may influence numerous processes within hair follicle environment leading to local autoimmune response resulting in an exacerbated hair loss.

This study has its limitations, such as relatively small number of participants. Future research including a greater sample size will allow the reassessment of the association between adipokines and alopecia areata.

## Conclusions

Patients with alopecia areata are characterized by an abnormal serum level of adipokines, particularly adiponectin and resistin. Adiponectin may be considered as a marker of severity of hair loss in alopecia areata. The results of the present study support the hypothesis that the impaired secretion of specific adipokines may play an important and complex role in the pathogenesis of alopecia areata and its continuity.

## Supplementary Information

Supplementary Information 1.

Supplementary Information 2.

Supplementary Information 3.

Supplementary Information 4.

Supplementary Information 5.

## References

[1] Fantuzzi G (2005). Adipose tissue, adipokines, and inflammation. J. Allergy Clin. Immunol..

[2] Mancuso P (2016). The role of adipokines in chronic inflammation. ImmunoTargets Ther..

[3] Stochmal A, Czuwara J, Zaremba M, Rudnicka L (2020). Altered serum level of metabolic and endothelial factors in patients with systemic sclerosis. Arch. Dermatol. Res..

[4] Ambroszkiewicz J (2018). Anti-inflammatory and pro-inflammatory adipokine profiles in children on vegetarian and omnivorous diets. Nutrients.

[5] Randhawa M (2009). Evidence for the ectopic synthesis of melanin in human adipose tissue. FASEB J..

[6] Page S, Chandhoke V, Baranova A (2011). Melanin and melanogenesis in adipose tissue: Possible mechanisms for abating oxidative stress and inflammation?. Obes. Rev..

[7] Slominski A, Paus R (1993). Melanogenesis is coupled to murine anagen: Toward new concepts for the role of melanocytes and the regulation of melanogenesis in hair growth. J. Invest. Dermatol..

[8] Slominski A, Wortsman J (2000). Neuroendocrinology of the skin. Endocr. Rev..

[9] Slominski AT (2012). Sensing the environment: Regulation of local and global homeostasis by the skin's neuroendocrine system. Adv. Anat. Embryol. Cell Biol..

[10] Slominski A, Wortsman J, Tobin DJ (2005). The cutaneous serotoninergic/melatoninergic system: Securing a place under the sun. FASEB J..

[11] Alkhalifah A (2013). Alopecia areata update. Dermatol. Clin..

[12] Safavi K (1992). Prevalence of alopecia areata in the First National Health and Nutrition Examination Survey. Arch. Dermatol..

[13] Mirzoyev SA, Schrum AG, Davis MDP, Torgerson RR (2014). Lifetime incidence risk of alopecia areata estimated at 2.1% by Rochester Epidemiology Project, 1990–2009. J. Invest. Dermatol..

[14] Rudnicka L, Waśkiel-Burnat A (2021). Systemic aspects of alopecia areata Comment to the article by Lai and Sinclair. J. Eur. Acad. Dermatol. Venereol..

[15] Paus R, Slominski A, Czarnetzki BM (1993). Is alopecia areata an autoimmune-response against melanogenesis-related proteins, exposed by abnormal MHC class I expression in the anagen hair bulb?. Yale J. Biol. Med..

[16] Waśkiel-Burnat A (2019). The value of dermoscopy in diagnosing eyebrow loss in patients with alopecia areata and frontal fibrosing alopecia. J. Eur. Acad. Dermatol. Venereol..

[17] Gilhar A (2001). Melanocyte-associated T cell epitopes can function as autoantigens for transfer of alopecia areata to human scalp explants on Prkdc(scid) mice. J. Invest. Dermatol..

[18] Rork JF, Rashighi M, Harris JE (2016). Understanding autoimmunity of vitiligo and alopecia areata. Curr. Opin. Pediatr..

[19] Olsen EA (2004). Alopecia areata investigational assessment guidelines—Part II. National Alopecia Areata Foundation. J. Am. Acad. Dermatol..

[20] Kibar M, Aktan S, Bilgin M (2015). Dermoscopic findings in scalp psoriasis and seborrheic dermatitis; two new signs; signet ring vessel and hidden hair. Indian J. Dermatol..

[21] Rudnicka, L. & Waśkiel-Burnat, A. Systemic aspects of alopecia areata Comment to the article by Lai and Sinclair. *J. Eur. Acad. Dermatol. Venereol*. 10.1111/jdv.16936 32918297

[22] Conic RRZ, Chu S, Tamashunas NL, Damiani G, Bergfeld W (2021). Prevalence of cardiac and metabolic diseases among patients with alopecia areata. J. Eur. Acad. Dermatol. Venereol..

[23] Karadag AS (2013). Insulin resistance is increased in alopecia areata patients. Cutan. Ocul. Toxicol..

[24] Shahidi-Dadras M, Bahraini N, Rajabi F, Younespour S (2019). Patients with alopecia areata show signs of insulin resistance. Arch. Dermatol. Res..

[25] Żółkiewicz J, Stochmal A, Rudnicka L (2019). The role of adipokines in systemic sclerosis: A missing link?. Arch. Dermatol. Res..

[26] Hutcheson J (2015). Adipokines influence the inflammatory balance in autoimmunity. Cytokine.

[27] Del Prete A, Salvi V, Sozzani S (2014). Adipokines as potential biomarkers in rheumatoid arthritis. Mediators Inflamm..

[28] Serarslan G, Özcan O, Okyay E, Ünlü B, Karadağ M (2020). Role of adiponectin and leptin in patients with alopecia areata with scalp hair loss. Iran. J. Med. Sci..

