# Supplementary material for: Adiponectin as a novel biomarker of disease severity in alopecia areata
Source: Sci Rep. 2021 Jul 5;11:13809. doi: 10.1038/s41598-021-92853-1 (PMC8257783; doi:10.1038/s41598-021-92853-1)
Supplement: Supplementary file 1 — Supplementary Information 1. [file 41598_2021_92853_MOESM1_ESM.docx]

**Supplementary figures legend:**

**Suppl. Fig. 1** A correlation between the serum concentration of resistin and the severity of alopecia areata.

**Suppl. Fig. 2** A correlation between the serum concentration of adiponectin and the duration of hair loss.

**Suppl. Fig. 3** A correlation between the serum concentration of resistin and the duration of hair loss.
