# Supplementary material for: Adiponectin as a novel biomarker of disease severity in alopecia areata
Source: Sci Rep. 2021 Jul 5;11:13809. doi: 10.1038/s41598-021-92853-1 (PMC8257783; doi:10.1038/s41598-021-92853-1)
Supplement: Supplementary file 2 — Supplementary Information 2. [file 41598_2021_92853_MOESM2_ESM.docx]

**Supplementary Table 1.** Differences in the serum concentrations of adiponectin and resistin between patients with active, stable and remitting alopecia areata.

| **Parameter**  **(Mean±SD)** | **Patients with active* AA**  **(n=21)** | **Patients with stable** AA**  **(n=25)** | **P-value** | **Patients with active* AA**  **(n=21)** | **Patients with remitting*** AA**  **(n=19)** | **P-value** |
| --- | --- | --- | --- | --- | --- | --- |
| **Adiponectin (ng/ml)** | 7177±3689 | 8433±4882 | 0.1275 | 7177±3689 | 7628±3463 | 0.9886 |
| **Resistin (ng/ml)** | 10.99±2.20 | 10.76±3.90 | 0.5235 | 10.99±2.20 | 11.46±5.25 | 0.5695 |

SD – standard deviation, AA – alopecia areata

* Active AA – an increase in total hair loss of more than 5% during 1 month prior to laboratory tests

** Stable AA – a change in total hair loss of less than 5% during 1 month prior to the laboratory tests

*** Remitting AA – a decrease in total hair loss of more than 5% during 1 month prior to the laboratory tests
