# Supplementary figures and images for: Adiponectin as a novel biomarker of disease severity in alopecia areata
Source: Sci Rep. 2021 Jul 5;11:13809. doi: 10.1038/s41598-021-92853-1 (PMC8257783; doi:10.1038/s41598-021-92853-1)

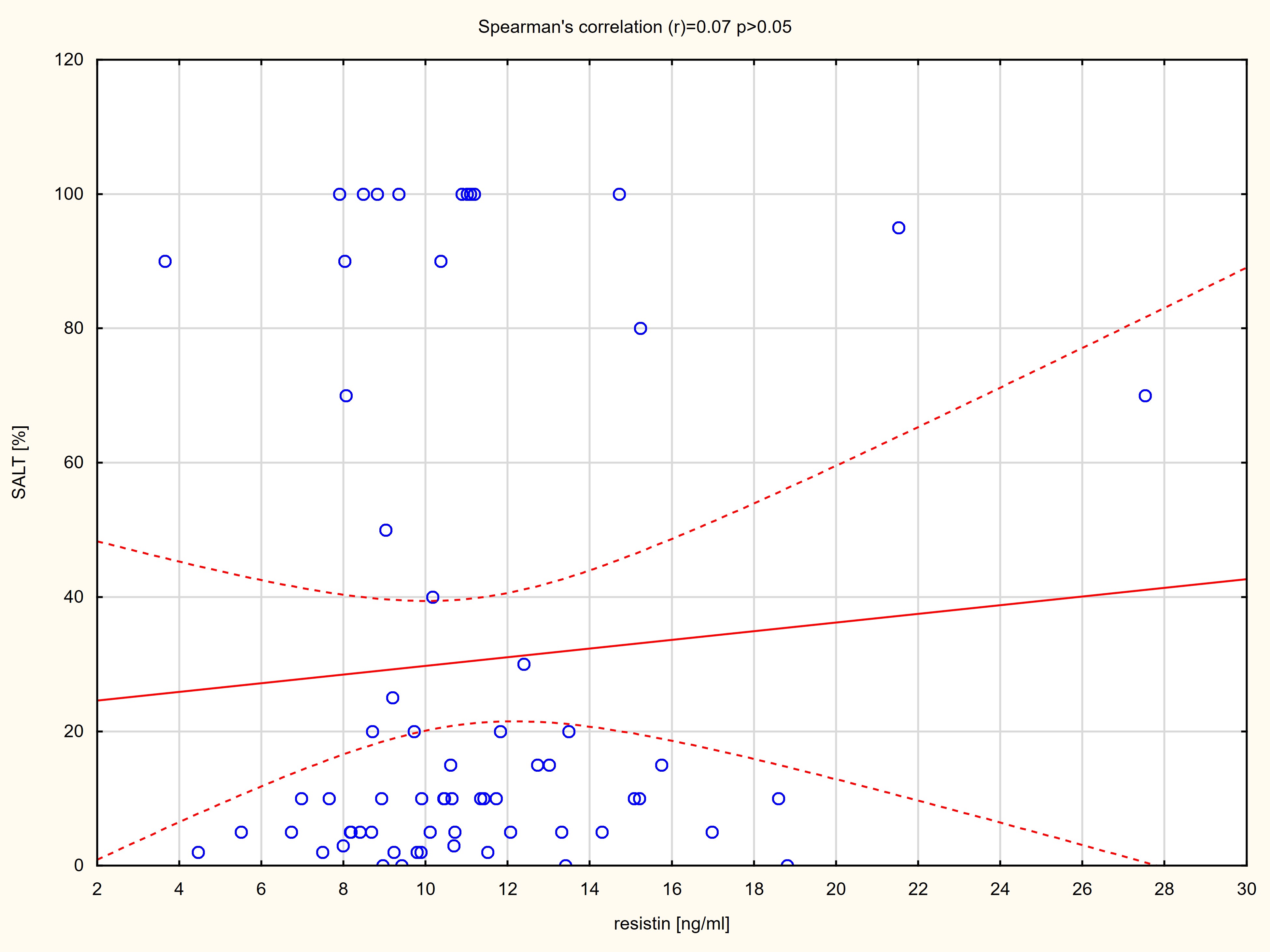

Supplement: Supplementary file 3 — Supplementary Information 3. [file 41598_2021_92853_MOESM3_ESM.jpg]

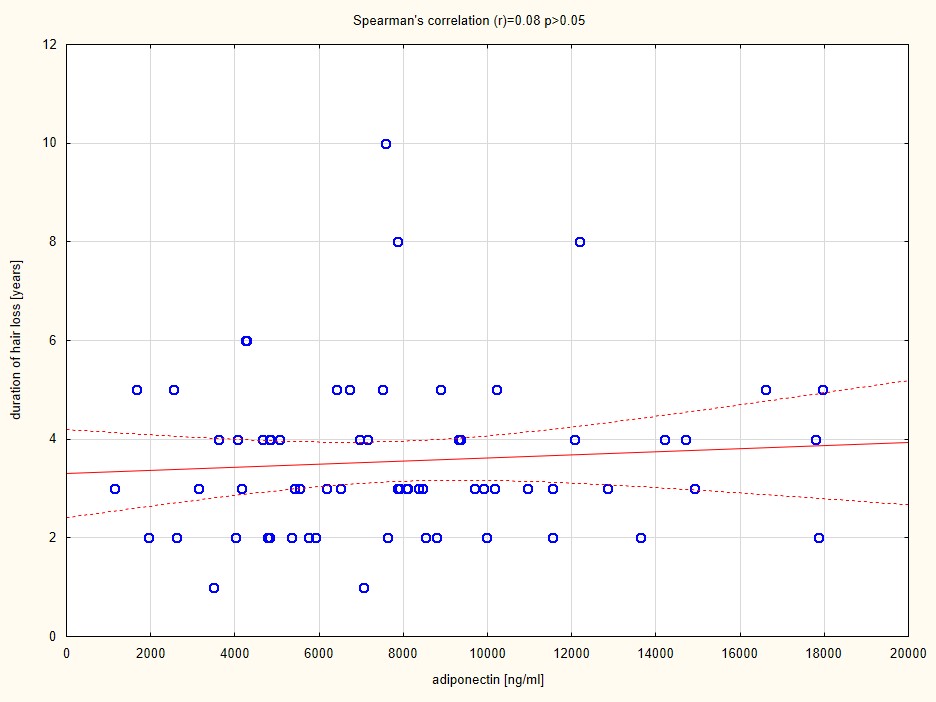

Supplement: Supplementary file 4 — Supplementary Information 4. [file 41598_2021_92853_MOESM4_ESM.jpg]

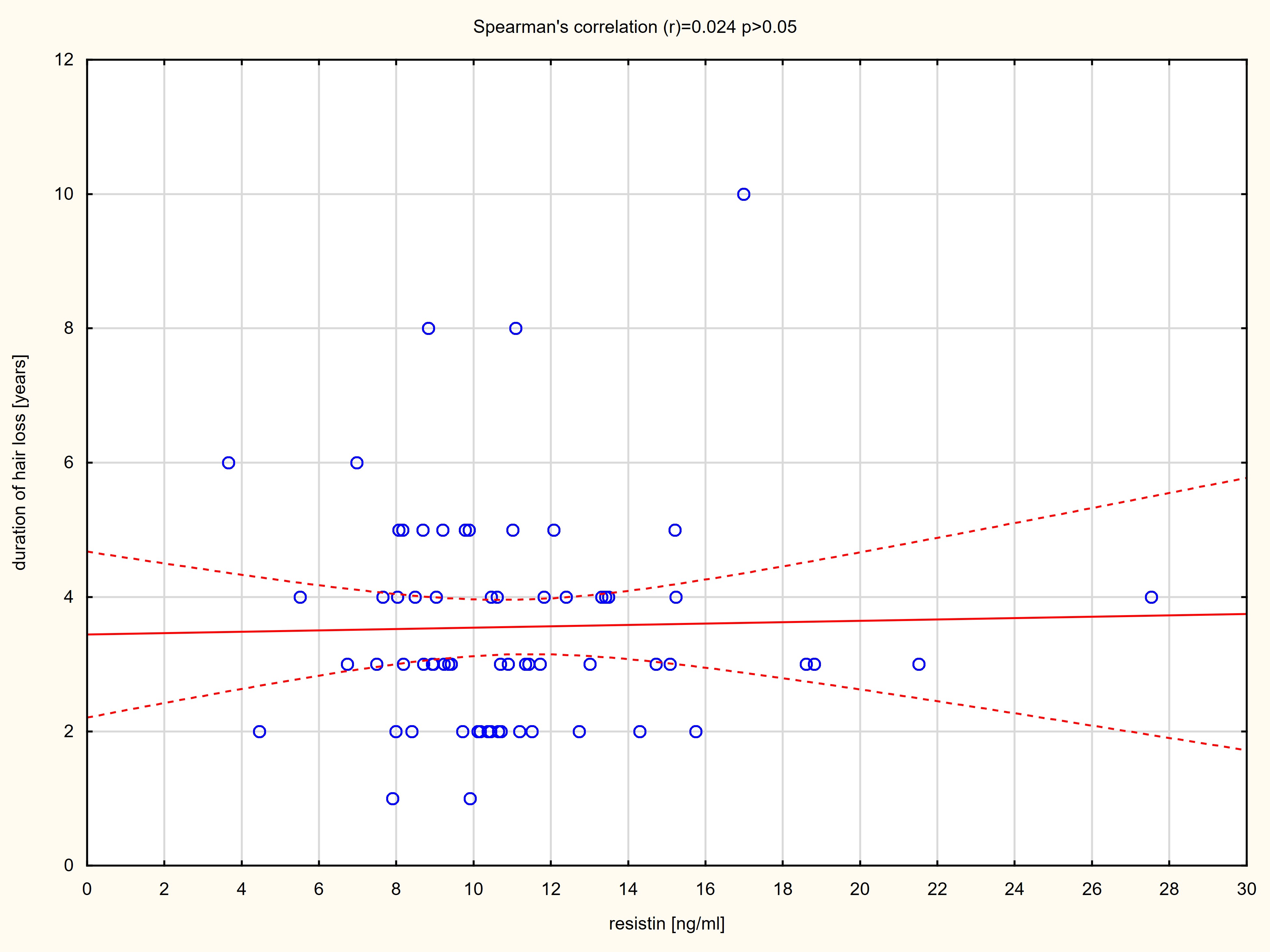

Supplement: Supplementary file 5 — Supplementary Information 5. [file 41598_2021_92853_MOESM5_ESM.jpg]
